# Preventing Nosocomial MDR-TB Transmission in sub Saharan Africa: Where Are We at?

**DOI:** 10.5539/gjhs.v5n4p200

**Published:** 2013-05-15

**Authors:** Sonia S. Menon

**Affiliations:** 1 International Centre for Reproductive Health, Ghent University, Ghent, Belgium; 2 London School of Hygiene and Tropical Medicine Alumni, London, United Kingdom

**Keywords:** WHO re-treatment protocols, nosocomial MDR-TB transmission, sub-Saharan Africa, infection control practices

## Abstract

**Background:**

In sub Saharan Africa, the cocktail of many advanced HIV-infected susceptible hosts, poor TB treatment success rates, a lack of airborne infection control, limited drug-resistance testing (DST) have resulted in HIV-infected individuals being disproportionately represented in Multi drug resistant Tuberculosis (MDR-TB) cases. The prevailing application of the WHO re-treatment protocol indiscriminately to all re-treatment cases sets the stage for an increase in mortality and MDR-TB nosocomial transmission.

**Method:**

A comprehensive search was performed of the Cochrane Infectious Diseases Group Specialized Register and Medline database including the bibliographies of the retrieved reference.

**Findings:**

The TB diagnosis paradigm which for decades relied on smear sputum and culture is likely to change with the advent of the point-of-care diagnostic, Xpert MTB/RIF assay. Until the new DST infrastructure is available, along with clinical trials for both, current and new approaches to retreatment TB in areas heavily affected by HIV and TB, there are cost effective administrative, environmental, and protective measures that may be immediately instituted.

**Conclusion:**

The severe lack of infection control practices in sub Saharan Africa may jeopardise the recent strides in MDR-TB management. Cost effective infection control measures must be immediately implemented, otherwise the development of further drug resistance may offset recent strides in MDR-TB management.

**Indiscriminate use of the WHO standardized retreatment protocol can lead to nosocomial transmission of MDR-TB by:**

-Precluding early diagnosis and prompt separation of patients who experienced treatment failure category and thereby more likely to have MDR-TB.

-Leaving patients from the treatment failure category in health establishments on ineffective standard retreatment regimen until the DST results are known.

-Targeting only patients who have had prior TB therapy, new severely debilitated TB patients having primary unrecognized MDR-TB may continue spreading resistant organisms.

## 1. Introduction

Standardized treatment regimens with first-line drugs are highly efficacious in drug-susceptible tuberculosis and fairly successful in isoniazid-only-resistant tuberculosis. Treatment standardization has also been advocated as a feasible and potentially effective approach for MDR-TB in low-income settings, where levels of resistance to second-line drugs are still low.

Every year, 10%–20% of people with TB in low- and middle-income countries are started on a standardized five-drug retreatment regimen as recommended by the WHO consisting of a three-month initial phase and a five-month continuation phase is still being applied to all re-treatment cases. Unlike treatment regimens for new TB patients, this regimen had never been evaluated for efficacy in randomized clinical trials or prospective cohort studies (López et al., 2011).

Re-treatment cases include cases of relapse, treatment after default and treatment failure. Treatment failure is defined as sputum smear positive at five months or later. “Treatment failure” has been associated with a high prevalence of drug resistance in an area of HIV prevalence, as illustrated in 57.1% of retreatment patients in KwaZulu-Natal exhibiting resistance compared to other treatment category (Schreiber et al., 2009).

MDR-TB defined as resistance to both isoniazid and rifampicin, can either be acquired due to poor adherence to treatment or primary when someone is infected with a strain which is already drug-resistant. The association between HIV infection and the development of MDR-TB has not yet been fully clarified. Some studies have shown increased rates of drug-resistant TB among HIV-infected individuals, whereas others have failed to confirm this finding (Suchindran, Brouwer, & Van Rie, 2009).

The short intervals of only 4 to 16 weeks for HIV-positive patients from MDR-TB diagnosis to death, despite receiving directly observed therapy with isoniazid, rifampicin, pyrazinamide, ethambutol, along with the high transmission rates from patients to health-care providers pose important public health problems (Daley et al., 1992).

Hospital outbreaks due to MDR-TB were reported in association with HIV-positive patients in the 1990s (Dooley, Jarvis, Martone, & Snider, 1992), across the world. Sub-Saharan Africa would seem to have all of the conditions for a “perfect storm” of HIV infection and MDR-TB, where re-treatment cases and new severely disabilitated TB patients often stay in the same ward.

According to the WHO XDR Summary report (2006), extensive drug related TB (XDR-TB), defined as MDR-TB plus resistance to fluoroquinolones and second-line injectable drugs, was found in 2006 KwaZulu Natal, South Africa. It spread rapidly among HIV immunocompromised patients and was associated with extremely high mortality (Gandhi et al., 2006). Recent milestones in diagnosis and treatment of MDR-TB along with newer WHO guidelines emphasizing DST for at least retreatment patients offer hope.

Firstly, this paper sets out to identify research gaps pertaining to each of the three WHO guidelines for TB case retreatment. Secondly, it will show how the application of the standard WHO retreatment regimen may exacerbate the inadequacies of the current first line administrative measures advocated to prevent nosocomial MDR-TB in TB clinics within high HIV prevalence settings. Thirdly, it will outline immediate cost-effective administrative, environmental, and protective measures that may be instituted to prevent nosocomial MDR-TB transmission in sub-Saharan African health care institutions. Finally, it will be argued that the dearth of infection control practices should be addressed, if the strides in MDR-TB management are to be sustained.

### 1.1 Method

The Medline database and the Cochrane Infectious Diseases Group Specialized Register, including the bibliographies of the retrieved reference were searched for the following words: WHO retreatment protocol; Multi drug resistant TB management; nosocomial Multi drug resistant TB transmission Africa; administrative control TB; environmental control TB; and personal protection TB.

## 2. WHO Retreatment Protocols

Management of patients who have been previously treated for TB has since long been a contentious issue. In 1991, the World Health Organization (WHO) recommended the use of the “category II retreatment regimen” for all patients with a prior history of TB treatment. The category II regimen added streptomycin to the first-line agents and extended treatment to 8 months. In 2006, these guidelines were fine-tuned and the WHO guidelines recommended DST prior to retreatment and treatment of confirmed treatment failures and suspected MDR-TB cases with region-specific standardized regimens. However, a lack of laboratory infrastructure for culture and DST in many settings with a high burden of tuberculosis resulted in widespread empirical use of the retreatment regimen. Coupled with intermittent retreatment adherence, standardized retreatment regimens may have inadvertently fuelled the emergence of MDR-TB.

This ensuing escalating problem of MDR-TB gave rise to new WHO international guidelines for retreatment of tuberculosis in 2010. According to these revised WHO guidelines, the surveillance and early diagnosis of MDR- TB should be ideally carried out by routine DST of all TB patients. Where the means do not allow individualized treatment of all new TB cases, standardized treatment, based on local resistance patterns determined from population-based anti-tuberculosis drug resistance surveillance should be administered. In settings where DST results are not yet routinely available, the empirical regimens should continue throughout the course of treatment.

### 2.1 WHO Standardized Retreatment Regimen

The WHO recommended standardized retreatment regimen protocol, without the use of routine drug susceptibility testing is suited to settings with homogeneous drug resistance patterns. As this strategy is less demanding in terms of tests and monitoring, it facilitates clinical management and is less laboratory-dependent. Annually more than 1 million people in over 90 countries are treated with the 8-mo regimen (2 mo of streptomycin [S], isoniazid [H], ethambutol [E], rifampicin [R], and pyrazinamide [Z]; 1 mo of R, H, E and Z; and 5 mo of R, H and E [2 SHERZ/1 RHEZ/5 RHE]) after failing, interrupting, or relapsing from prior treatment. WHO (2009). Unlike treatment regimens for new TB patients, this retreatment regimen has never been subjected to randomized controlled trials (RCTs) or prospective cohort studies.

Although, WHO surveillance data suggest that the retreatment regimen is successful in about 70% of patients (WHO, 2009), for almost two decades, country and multi-country studies have gathered unfavourable evidence (Kritski et al., 1997). The findings of a recent prospective study in Uganda provide evidence that the standard retreatment approach to TB, as implemented in low- and middle-income settings, was inadequate. Even after cure, very high rates of recurrence in TB patients with MDR-TB at baseline. Although a 70%–80% of patients with and without HIV co-infection were found to have a successful treatment outcome, 20% of HIV-uninfected and 26% of HIV-infected patients had an unsuccessful treatment outcome. HIV-infected individuals were also more likely to die than HIV-uninfected individuals. Despite the findings that inadequate regimens amplify drug resistance, the standardized retreatment regimen is still generally the mainstay of national programs in resource-limited settings (López et al., 2011). This is also likely to increase mortality and MDR-TB nosocomial transmission.

New Fluroquinolones has revived hope for combatting emerging resistant strains. Fluroquinolone may reduce the current recommended duration of treatment for MDR-TB which stands at 18–24 months. A recent observational study in Bangladesh showed that a short, standardized treatment regimen based on a fourth-generation fluoroquinolone combined with other second-line drugs and supplemented by potentially still active first-line drugs was highly effective and safe in mostly HIV-negative patients without a history of prior treatment with second-line drugs (Van Deun et al., 2010). However, its efficacy as well as recurrence rates has yet to be assessed in a randomized control trial (RCT) for both HIV-negative and HIV-positive patients.

### 2.2 WHO Region-Specific Standardized Regimens

An updated 2006 WHO guidelines recommended DST prior to retreatment and treatment of confirmed treatment failures and suspected MDR-TB cases with region-specific standardized regimens. This guideline proposed an empiric alternative based on local resistance patterns determined from well-performed population-based anti-tuberculosis drug resistance surveillance. However, few high burden countries met the WHO standards of maintaining at least one culture laboratory for 5 million people and one facility capable of conducting any susceptibility testing per 10 million and some even lack a National Reference Laboratory (NRL) to perform the most basic surveillance (WHO, 2009). By 2010, only South Africa had the capacity to diagnose MDR-TB and XDR-TB (USAID, 2010). In 2012 a second supranational laboratory was also established in Uganda, facilitating testing MDR-TB and XDR.TB testing for the entire East African region.

The design of region-specific standardized regimens is contingent upon the epidemiological context, the efficacy of the program, and the available resources. The design of region-specific standardized regimens depends on the epidemiological context, the program's performance, and the means available. In a follow up study of MDR-TB patients in Nepal, Pushpa Malla et al. (2009) observed that when a standardized treatment regimen for MDR-TB is based on good drug resistance surveillance data, the cure rates at 70% could be in line with individualized treatment regimens based on DST results. In Peru a cohort of MDR-TB patients treated with a fully standardized regimen had cure rates of 57% of the patients being cured (Suárez et al., 2002), probably due to the improper consideration of the underlying resistance patterns to first- and second-line drugs in the population (Pushpa Malla et al., 2009). Also, in Cameroon, the number of tuberculosis patients reported with resistance to first-line anti-tuberculosis drugs after a standardized retreatment regimen proved to be unacceptably high among standard regimen failure cases and following the establishment of a supervised Regional TB Reference Laboratory with culture capacity (Noeske, Voelz, Fon, & Foe, 2012).

### 2.3 New WHO Revised Guideline

According to the revised 2010 WHO guideline, the surveillance and early diagnosis of drug-resistance in TB is ideally carried out by routine DST of all TB patients. Where the means do not permit systematic assessment of all cases with a new episode of TB, alternatively, at least patients known to be at higher risk of carrying drug-resistant strains such as previously treated patients are to be assessed systematically.

In an individual patient data meta-analysis of observational data, on patients in whom DST was routinely performed, it was found that improved MDR-TB treatment success and survival were associated with the use of certain fluoroquinolones, ethionamide, or prothionamide, and greater total number of effective drugs. This offers hope and underscores the future need to undertake RCTs to optimize MDR-TB treatment in both HIV-negative and HIV-positive patients, when DST will be more routinely performed in Sub-Saharan Africa.

Noeske et al. (2012) undertook a pilot programme in Cameroon aimed at performing routine DST among previously treated pulmonary TB under programmatic conditions. Findings illustrated the feasibility of WHO recommended routine DST in retreatment patients. However, lack of rapid diagnostic tools and reliance on solid media cultures, led to results only being known at the 5-month control period.

Making this new guideline feasible, a recent breakthrough in the TB diagnostic landscape is the advent of the point-of-care diagnostic, MTB/RIF assay (Xpert). A meta-analysis published in 2013 indicated that it was both sensitive and specific as an initial diagnostic test for TB detection and rifampicin resistance detection in patients having TB, MDR-TB, or HIV-associated TB.

A recent study carried out by Palacios et al. (2012) also showed how early ART is likely to become a key component of effective MDR-TB management in co-infected individual. This would be an argument in favour of the WHO 2009 guideline recommending earlier ART initiation at 350 cell/mm3 in sub Saharan Africa, which due to the absence of large prospective trials, gives rise to many uncertainties (Menon, 2010)

## 3. Nosocomial transmission in sub Saharan Africa

As high rate of HIV co-infection is one of the major reasons for the high mortality rate among drug-susceptible TB patients in sub-Saharan Africa (Harries, Hargreaves, Gausi, Kwanjana, & Salaniponi, 2001), it is likely that new TB patients hospitalized are at an advanced immunosupression stage of HIV. This would make them particularly prone to circulating MDR-TB strains coming from re-treatment cases. Likewise, MDR-TB patients may incur a high risk of becoming multiply infected.

In an assessment of organizational measures to prevent nosocomial tuberculosis in health facilities involved in TB management of 4 sub–saharan countries in 2010, Robert et al. (2013) found that none had a TB infection control plan, and only 5.2% provided education for staff about nosocomial TB. Overall, 48.3% of the facilities performed triage of suspected TB cases on hospital arrival, 89.6% provided education for TB cases on cough etiquette, 20.0% segregated smear-positive TB cases, and 15.7% segregated previously treated cases. A total of 15.5% of the facilities registered TB among staff, for a global prevalence rate of 348 cases per 100,000 staff members. In Nigeria, in an assessment of TB infection control practice in health care facilities implementing joint TB/HIV activities, Ogbonnaya, Chukwu, Uwakwe, Oyibo and Ndukwe (2011) found that only in 2 (16.7%) facilities, were patients asked questions concerning cough while obtaining a hospital card, and only in one of them was the duration of the cough also ascertained. However, in none of these two facilities were deliberate steps taken to fasten the administrative processes in order to investigate and confirm or rule out TB from such patients. A descriptive study also showed limited infection control practices in clinics in KwaZulu-Natal, South Africa. Naidoo, Seevnarain and Nordstrom (2012) found that of 51 clinics, 11 (22%) had infection control policies, 13 (26%) triaged coughing patients and 16 (31%) had a dedicated nurse and a dedicated consulting room for treating tuberculosis (TB) patients.

### 3.1 Administrative Infection Control Measures

Administrative control measures are the first line of defense against TB transmission and are meant to reduce patient exposure to infectious particles and decrease staff exposure. First line administrative measures include early diagnosis of potentially MDR- TB patients, prompt separation or isolation of MDR- TB patients, and the prompt initiation of appropriate anti-tuberculosis treatment.

#### 3.1.1 Early Diagnosis of Potentially MDR-TB Patients in High HIV Prevalence Setting

With the application of the original WHO retreatment protocol, patients may be misclassified. Severely debilitated new TB patients having unrecognized MDR-TB may not receive adequate therapy and continue spreading resistant organisms in health establishments. Also, non MDR-TB cases may be included among re-treatment cases. Non MDR-TB patients placed in the suspected MDR-TB category may include patients with advanced immunosuppression, who may be prone to recurrent infections with non-tuberculous mycobacteria (NTM), which can be mistaken for TB treatment failure (Churchyard, Kleinschmidt, Corbett, Mulder, & De Cock, 1999).

#### 3.1.2 Baseline Triage and Separation Strategy

An effective baseline triage and separation strategy will vary according to HIV prevalence. An effective community-programme based on a baseline triage and separation strategy has been implemented in Haiti depending on smear results and HIV status (Institute of Medicine, 2004). Whilst this strategy consisting of establishing very basic isolation rooms on smear results and HIV is imperfect because it does not preclude sputum smear-positive patients from multiple infecting one another, re-infecting one another, and from unsuspected cases from posing a threat, it was deemed highly effective if in settings where currently no transmission control exists. According to USAID (2010), this strategy was effective in Haiti, where 2.2 % of adults are estimated to be HIV-positive.

However, this strategy may not be feasible in other settings with a higher prevalence of TB/HIV co-infection. The safest but far more expensive solution to reduce the risk of nosocomial transmission in a high HIV prevalence setting would be an individual room for each smear-positive patient (AIDSMAP, 2008). As this lacks feasibility, clinicians must be trained with the aid of clear guidelines and protocols to identify patients at high risk for MDR-TB, segregate them from both non MDR retreatment patients and other TB patients (Kwonjune et al., 2009).

#### 3.1.3 Prompt Initiation of Appropriate MDR-TB Treatment

Prompt initiation of appropriate MDR-TB treatment purports to bring clinical improvements to the patient as well as curb nosocomial transmission. In HIV-positive patients constitutes a challenge and requires a high index of clinical suspicion, to initiate second-line TB drugs empirically before the results of DST are known (Cohn, 1994).

Moreover, the high prevalence of HIV, both in patients, and health care workers (HCW) also requires specific administrative infection control measures. The risk of nosocomial transmission of MDR or XDR TB cannot be adequately addressed lest the seropositivity of all TB patients and HCWs is known. With the impact that the HIV epidemic have had on the efficacy of sputum examinations and active radiological screening programmes, fewer TB cases will be detected through an annual active case finding programme (Churchyard et al., 1999). HIV-positive HCWs may need to be targeted more frequently for MDR-TB screening to improve case detection and detect early and potentially less extensive pulmonary disease.

Whilst prophylaxis for latent TB may prevent active TB, there is no consensus on optimal treatment for persons with known exposure to MDR-TB. A systematic review of treatment for latent infection with TB in persons at risk for MDR-TB found only two cohort studies, which met the inclusion criteria. The earlier retrospective cohort study conducted by Schaaf et al. (1999) found isoniazid not to be effective (RR 0.46, 95% CI 0.07–2.32) while a later prospective cohort study conducted by Schaaf et al. (2002) found individualised tailored treatment with high-dose isoniazid, pyrazinamide, ethionamide and/or ethambutol and/or ofloxacin to be effective (RR 0.20, 95% CI 0.04–0.94). Despite the lack of consensus, the ATS/CDC in 2000 recommended that a combination of Pyrazinamide and Ethambutol along with either Pyrazinamide or Fluoroquinolone be administered to immunocompetent contacts for 6 months and 12 months to immunocompromised contacts.

If an infection control plan is drawn up, it needs to consider that treating Latent TB Infection (LTBI) or latent MDR-TB or in HIV-infected persons prior to the initiation of HAART clinical TB will also “unmask” undiagnosed TB via reconstitution (CDC, NIH, & HIVMA/IDSA, 2008). Inadvertently, this may potentially increase the risk of transmission. Once there is consensus, it would be advisable that prophylaxis be given to all HIV-positive HWC before initiation of HAART (Pettit et al., 2010) if active disease can be discarded on radiological, clinical and bacteriological grounds.

Compounding the high risk of MDR-TB nosocomial transmission due to the indiscriminate use of the WHO standardized retreatment protocol, is the lack of genotype tools. This precludes the undertaking of a pivotal molecular epidemiological investigation alongside a conventional epidemiology study. Similar Restriction Fragment Length Polymorphism (RFLP) patterns using IS6110 as a probe with identical resistance profiles would be indicative of a potential nosocomial MDR-TB outbreak, instead of a reactivation of strains acquired in the more distant past.

#### 3.1.4 Immediate Available Administrative Infection Control Measures

Inadequacies pertaining to the implementation of first line administrative infection control measures, lack of genotype tools, as well as clear guidelines to identify retreatment patients at risk of MDR-TB, all beg the need for other immediate more affordable “second line” administrative measures. The best makeshift option in a setting of high HIV prevalence would be to segregate retreatment patients from all other patients. Also, it should be recognised that hospitalising TB patients, whether they are drug-sensitive or drug-resistant for extended periods of time makes them more vulnerable to reinfection with MDR and even XDR-TB strains. Therefore, discharging patients without co-morbidities for ambulatory treatment just after sputum conversion may be an effective measure to reduce the risk of nosocomial transmission.

These measures could be accompanied by behaviour-change campaigns in TB clinics promoting a better cough etiquette and respiratory hygiene for patients, HCWs and visitors. If physical barriers, such as a piece of cloth, a tissue or surgical mask are available, the campaign should also include proper disposal as an integral part of respiratory hygiene practice (Kellerman, Tokars & Jarvis, 1998).

If no physical barriers are available, best practice suggests that the mouth and nose should be covered with the bend of the elbow or hand, which must then be cleaned immediately (AIDSMAP, 2013).

Furthermore, HIV-positive HCWs may reduce occupation health risks by minimizing their exposure to suspected MDR-TB cases and to patients undergoing procedures to stimulate coughing. In the Nigeria study, none of the facilities had health education materials, including posters and leaflets educating patients and reminding HCWs of the possibility of TB transmission within the health care setting (Ogbonnaya et al., 2011).

However, the implementation of these “second line” administrative control measures have unfortunately little chance of being implemented in health care facilities implementing joint TB/HIV activities, where a basic immediate triage of coughing patients does not even take place.

### 3.2 Environmental Measures in High HIV Prevalence Settings

The second level of the hierarchy is the use of engineering controls to prevent the spread and reduce the concentration of infectious droplet nuclei (CDC, 2005). Well-maintained negative-pressure isolation facilities, which constitute optimal standard of care for infectious respiratory patients, lack feasibility in sub Saharan TB clinics.

Instead, natural ventilation which is maintenance free is particularly suited to tropical resource-limited settings (Escombe et al., 2010). Natural ventilation was shown to be very effective at reducing the risk of airborne contagion especially in older, spacious facilities with high ceilings and large windows on more than one wall, even in fairly wind-still condition (Escombe et al., 2010).

In South Africa, an epidemiological mathematical modelling study showed that improvements to natural ventilation could prevent 33% of future XDR-TB cases (Basu et al., 2009). Whilst affordable, natural ventilation may not reliably grant the protection that is required. A disadvantage of natural ventilation is the difficulty in controlling direction of airflow due to the absence of negative pressure. Contamination of corridors and adjacent rooms may therefore be a risk, particularly on completely still days (Escombe et al., 2010).

Alternatively, mechanical ventilation systems are also often used, as they overcome many of the problems associated with natural ventilation. However, mechanical ventilation is not without its disadvantages. Poorly designed or maintained systems facilitate pathogen distribution systems as illustrated by such failings in numerous TB outbreaks, even in the developed world. Modern wards with low ceilings and small windows were associated with higher risk, and mechanically ventilated rooms with sealed windows had even greater risk, despite being ventilated optimally according to guidelines (Escombe et al., 2010).

There has been a revival of interest in ultraviolet germicidal irradiation (UVGI), designed to reduce the microbial level in the room air and strategically positioned to protect vulnerable patients and staff. Confirmatory evidence of UVGI efficacy against human-generated TB in Peru and in South Africa highlights its potential role in facilities that provide MDR/XDR treatment in tropical resource-poor settings as an adjunct to natural ventilation at night when windows are closed due for security reasons or when climatic conditions are unfavourable (Xu et al., 2009).

The potential efficacy of the UVGI has been proven in Lima, Peru, where relative humidity is generally 70%–90%, and the hospital rooms studied not air conditioned. Studies of health clinics in higher tropical humid environments should also be undertaken as UV germicidal efficacy is adversely affected by increasing relative humidity, particularly above 50%–60% (Xu et al., 2009).

Moreover, engineering specifications for a given room application remain elusive (Nicholas, 2010). Guidelines for the design and installation of upper room UVGI systems have been published, but are currently based more on common sense and historical practice than on actual evidence (Xu et al., 2009). There is a need for both, basic engineering studies to provide evidence-based engineering specifications as well as simple UV fixtures for low-resource settings (Nicholas, 2010). In addition, experts on UVGI application in sub Saharan settings are needed, who in turn will be able to impart local engineers with the technical expertise required for maintaining the equipment.

#### 3.2.1 Immediate Available Environmental Measures

Installing the UVGI without all these enabling conditions may not be cost effective and put the few resources available for administrative control and personal equipment measures in jeopardy. An immediate option in sub-Saharan TB clinics would be to separate well -ventilated area by using windows and doors to direct air flow to the outside environment and not vice versa in wards for suspected MDR-TB cases and other wards (Xu et al., 2009). Research demonstrated that protective rates of ventilation are still achievable with windows only partially open (Escombe et al., 2010).

### 3.3 Personal Respiratory Protection

As current options for personal prophylaxis for HCWs at increased risk for MDR-TB remains limited due to lack of consensus on optimal treatment for persons with known exposure to MDR-TB, personal respiratory protection for HCWs is a mainstay.

The 1999 WHO recommendations consider personal respiratory protection as the third line of defense for TB control when TB risk cannot be adequately reduced by administrative and engineering controls. These include respiratory masks, which contrary to facemasks prevent contamination by small particles. If fitted and used properly to prevent face seal leaks, a respirator (U.S certified N95 or EU certified FFP2 respirator masks) has been found to greatly reduce the chance that inhaled air will contain infectious tubercle bacilli (Bock, Jensen, Miller, & Nardell, 2007).

However, respirators that are not worn as recommended or cared for in a manner that maintains their integrity afford little protection (Biscotto, Pedroso, Starling, & Roth, 2005). One study in a 150-bed public Brazilian hospital that serves as reference hospital for tuberculosis patients showed that facial-seal leakage was observed in 39% of HCWs due to failure to wear the mask with a tight facial fit as directed (Biscotto et al., 2005). This underscores the need for hospital to train its personnel on its proper use and removal before allocating the limited resources on respirators when planning TB control programmes. Moreover, this study illustrated that respirator use as a sole control measure was inadequate in any setting and is not cost- effective in resource-limited settings (Biscotto et al., 2005).

Disposable N95 respirator masks for prolonged periods of time may be an economically more suitable option. Although filtering facepiece respirators have been reused during public health crises in resource-limited settings, the safety and efficacy of this approach has yet to be confirmed. Suggestions for the duration of disposable N95 respirators masks have varied from one week to a month in some African countries (OSHA, 2009).

Decontaminate filtering facepiece respirators may create a health hazard for the user and it may render the respirator ineffective in providing respiratory protection. Reuse by HCW may increase the potential for contamination through contact transmission and lead to transmission of an infectious microorganism to the patient or to oneself. Therefore, the risk of contaminating the inside with microorganisms of the respirator through improper handling must be weighed against the need to provide respiratory protection (OSHA, 2009).

#### 3.3.1 Immediate Alternative Personal Protection Measures

The lack of robust evidence base regarding the effectiveness of personal protective technologies in resource-poorer clinical settings, warrants that other immediate more cost effective personal respiratory protection be integrated for optimal protection.

In sub-saharan Africa, as respirators may be relatively costly to implement, it may not be feasible for all HCWs to be equipped with a N95. In an assessment of 51 primary health care clinics in Kwala et al. (2012) found that eleven (22%) of 51 clinics had N95 masks available for staff use. In Nigeria, none of the health care establishments involved in HIV/TB activities were using any respiratory control measures (Ogbonnaya et al, 2011).

A more cost effective strategy would be to prioritize HIV-positive Health Care Workers (HCWs) as well as HCWs in the vicinity whenever high-risk aerosol generating procedures are performed and those in charge of suspected MDR-TB patient management. While waiting for available data on decontamination of respirators to formulate evidence based recommendations, hand washing before and after touching the respirator CDC 2012 may be emphasized.

In settings where N95 respirators may not be affordable, surgical masks may prevent the inhalation of large airborne contaminants. A study conducted in specialized airborne infections research facility in South Africa, revealed that when infectious patients with MDR-TB wore surgical face masks while they were hospitalized, the masks helped decrease the transmission of MDR tuberculosis by 50 % compared to when the patients did not wear face masks (Dharmadhikari et al., 2011). Even a surgical mask that filters only 50% of inhaled particles would have the equivalent effect, in theory, of a doubling of room ventilation, and at a fraction of the cost (Bock et al., 2007).

The purchase of surgical masks may be an alternative for HCW likely to have lower MDR-TB exposure. An alternative would be to equip all HCWs with surgical masks, should there be no resources, nor facilities for implementing a N95 respiratory infection control system for higher at risk HCW. Although this could imply that individual subjects may not always be optimally protected, from a public health point of view, it would still decrease transmission (Van der Sande, Teunis, & Sabel, 2008).

## 4. Discussion

In light of recent XDR-TB outbreaks, future point of care diagnostics may also need to carry out DST for second-line anti-TB drugs to all patients with confirmed MDR-TB. Meanwhile, there is an urgent need for clear clinical guidelines and protocols to identify patients at high risk for MDR-TB in both re-treatment cases and new TB cases. Another research area in the TB control programme that needs to be addressed is treatment of latent MDR-TB. A systematic review on the treatment for latent infection with TB in persons at risk for MDR-TB highlighted the limitations in terms of both quantity and quality and underscored the need for RCTs to be undertaken to assess the effectiveness of treatment.

Moreover, given the high proportion of patients with NTM in sub-Sahara who end up being treated with regimens for MDR-TB and where mycobacterial identification is too expensive to be routine practice, international guidelines are also urgently needed for NTM case management (Nathanson et al., 2004).

However, since Minick, Castro, Harrington, Sacks and Burman (2007)'s call to start exploring shorter treatment for retreatment cases in the fluoroquinolone era, a potential breakthrough has been made in MDR-TB management. Provided that findings from the landmark study in Bangladesh in a mostly HIV-negative setting can be replicated in populations with high HIV prevalence and safely combined with ART with less intensive patient care, this would be a breakthrough in MDR-TB management in Sub Saharan Africa. Concomitantly, the wide-scale roll out of ART for symptomatic HIV patients and to an increasing number of HIV patients at a higher CD4 count may also contribute to enhancing MDR-TB management.

Albeit the MDR-TB management landscape has recently been granted a boost, which as a corollary may reduce the duration of infectiousness and the ensuing risk of nosocomial MDR-TB transmission, infection control practices existing in clinics in high TB and HIV burden settings remain limited. Descriptive studies in sub Saharan Africa have shown that often infection control policies, along with a basic triage of coughing patients are inexistent. Failure to prevent MDR-TB and XDR-TB nosocomial transmission cases in this setting may in turn also tangentially increase community MDR-TB transmission.

Provided that retreatment patients are isolated from other new TB patients, or let alone immediate triage of coughing patients takes place, there is a synergistic combination of immediate cost-effective nosocomial infection control strategies, which may be put in place that will not divert resources from the future DST scale up of retreatment cases and infrastructure. Alternatively for preventing escalating nosocomial MDR-TB transmission, an innovative community-based MDR-TB treatment models may be explored. In Lesotho, a community-based MDR-TB treatment model that involved patients in stable clinical condition showed that it may be feasible to provide clinically effective and cost-effective care in a setting where infrastructure for community is scarce (Seung et al., 2009). Before replicating this model in other HIV prevalence settings, aspects of community-based infrastructure needed to be strengthened should be identified in order to yield similar successful results in a different setting.

In the long term, barriers to the widespread use of genotyping in tuberculosis control programmes must be addressed. Given the complexity of IS16110 based genotyping, more rapid genotyping alternatives, such as Spoligotyping and MIRU should be available so that population-based surveillance genotyping can be incorporated into routine tuberculosis control to identify outbreaks (Barnes & Cave, 2003).

## 5. Conclusion

Changes in the traditional paradigms of TB diagnosis and MDR-TB management may lead to less bleak perspectives for nosocomial MDR and XDR TBtransmission prevention in Sub Saharan Africa.

The fluoriquinolone era brings forth chances of cure for MDR-TB cases. Also, new innovative point of care diagnostic tools may in the future enable all re-treatments to undergo DST, provided there is a concurrent commitment to develop the infrastructure needed to support this expansion. These changes herald better perspectives for nosocomial MDR and XDR TB transmission prevention in Sub Saharan Africa, where infection control practices in clinics are very limited.

However, the still prevailing application of the WHO re-treatment protocol indiscriminately to all re-treatment cases makes nosocomial transmission of MDR-TB all the more of a pressing public health issue. In light of this pending global public health catastrophe, certain cost-effective administrative, environmental and personal respiratory measures can readily be instituted.

It is imperative that the latest breakthroughs and the ones to come in MDR-TB management do not undermine efforts to address the prevailing lack of basic infection control practices. Tuberculosis infection control operational research in infection control in sub-saharan Africa should not be neglected. In so doing, it may inadvertently encourage the development of further drug resistance which would undermine the recent promising breakthroughs.
